# Zingerone Alleviates Acetaminophen‐Induced Liver Damage by Regulating Oxidative Stress, Inflammation, Apoptosis, Endoplasmic Reticulum Stress, and Autophagy

**DOI:** 10.1002/jbt.71045

**Published:** 2026-07-21

**Authors:** Serpil Aygörmez, Mustafa Makav, Sevda Eliş Yıldız, Elif Dalkılınç, Mushap Kuru, Şaban Maraşlı

**Affiliations:** ^1^ Department of Biochemistry Faculty of Veterinary Medicine, Kafkas University Kars Türkiye; ^2^ Department of Physiology Faculty of Veterinary Medicine, Kafkas University Kars Türkiye; ^3^ Department of Midwifery Faculty of Health Sciences, Kafkas University Kars Türkiye; ^4^ Department of Biochemistry Faculty of Veterinary Medicine, Atatürk University Erzurum Türkiye; ^5^ Department of Obstetrics and Gynecology Faculty of Veterinary Medicine, Kafkas University Kars Türkiye

**Keywords:** acetaminophen, endoplasmic reticulum stress, liver damage, oxidative stress, zingerone

## Abstract

The aim of this study is to investigate the protective effect of zingerone (ZNG), an antioxidant agent, against acetaminophen (APAP)‐induced liver damage, which is used as an analgesic and antipyretic. For this purpose, twenty‐eight male rats were divided into four groups: control, ZNG, APAP, and APAP + ZNG. ZNG was administered orally for 7 days, followed by a single dose of APAP on the 7th day. At the end of the study, biochemical, molecular, and immunohistochemical analyses were performed on the liver tissue. According to the data obtained, APAP was found to trigger oxidative stress, inflammation, autophagy, apoptosis, endoplasmic reticulum stress, autophagy, and heat shock proteins in liver tissue. On the other hand, it has been observed that after ZNG treatment, the activities of antioxidant enzymes SOD, GPx, and CAT increased, MDA levels, a significant indicator of lipid peroxidation, decreased, and GSH stores were replenished. Following ZNG treatment, apoptosis was attenuated, resulting in decreased mRNA transcript levels of Bax, caspase‐3, and increase in Bcl‐2 levels. ZNG treatment resulted in a decrease in endoplasmic reticulum stress, inflammation, autophagy mRNA transcript levels. In addition, a decrease in heat shock proteins was detected. In conclusion, it has been observed that ZNG may be a significant protective against APAP liver damage.

## Graphical abstract

Mechanism of action of zingerone on acetaminophen‐induced liver damage.

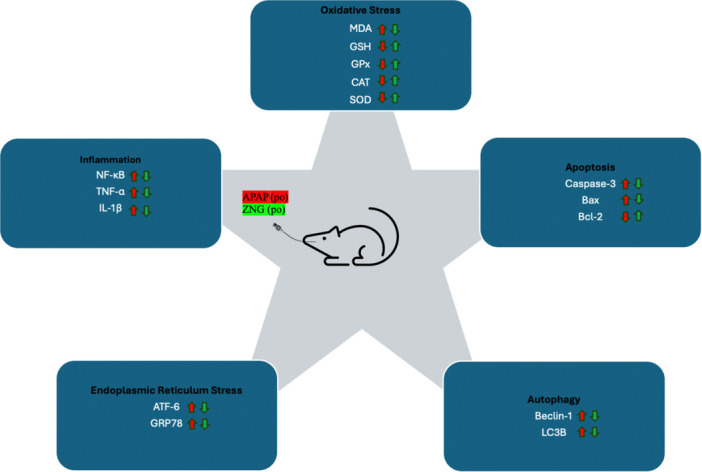

## 1 Introduction

The liver, the most important organ of the body, performs its biological functions during the metabolism of substances such as lipids, proteins, carbohydrates, and drugs [1]. Liver diseases seriously impact public health, becoming a global concern and a problem worldwide [1, 2]. Acetaminophen (APAP) is frequently used as an antipyretic and analgesic [3, 4]. While the Food and Drug Administration recommends APAP at 2000 mg or less for individuals with chronic alcohol consumption and liver disease, they have stated that a dose of 4000 mg is safe across a wide therapeutic range [5]. The safety and efficacy of the drug depend on liver function [2]. In APAP overdose, the amount in the blood increases, causing liver damage in addition to symptoms such as jaundice [5]. In the liver, the vast majority of APAP is metabolized into non‐toxic metabolites such as glucuronide and sulfate, while a small portion is converted into a toxic metabolite, N‐acetyl‐p‐benzoquinone imine [6]. High doses of APAP intake lead to the excessive production of N‐acetyl‐p‐benzoquinone imine, which in turn causes significant glutathione (GSH) depletion. It also causes covalent binding of sulfhydryl groups in proteins, leading to the production of highly reactive metabolites [7, 8]. GSH depletion results in oxidative stress due to an imbalance between antioxidants and an increase in reactive oxygen species [9]. The resulting oxidative stress leads to liver damage by producing mitochondrial peroxynitrite and proinflammatory cytokines such as tumor necrosis factor alpha (TNF‐α) [6]. Disruption of homeostasis triggers apoptosis, leading to dysfunction. In recent years, natural substances have attracted great interest in the treatment of liver disease [7]. For this reason, naturally occurring compounds with antioxidant properties are needed in APAP ‐induced liver damage [10].

Flavonoids, which exhibit pharmacological and biological properties in different cell types, are plant antioxidants [11]. Zingerone (ZNG), a flavonoid, is the bioactive component of ginger [11, 12]. ZNG, exhibiting properties such as antioxidant and hepatoprotective effects, possesses various pharmacological characteristics [13]. It also exhibits therapeutic properties in many tissues, including the liver [14, 15]. In addition, it also has effects on apoptosis, oxidative stress, cell differentiation, and proliferation [12]. In addition to these properties, it affects many signaling pathways involved in metabolic pathways [11]. The hydroxyl and methoxy groups in the chemical structure of ZNG enhance its free radical scavenging ability. It also disrupts oxidative chain reactions [16]. Due to its lipophilicity, rapid elimination, limited physicochemical stability, slow absorption, low bioavailability, low solubility in gastrointestinal fluid, and susceptibility to enzymatic degradation in the gastrointestinal system, ZNG requires the administration of significant oral doses to manage various conditions [17].

Recent studies on APAP have shown that APAP plays a role in liver damage. It is not fully known on which of these known mechanisms ZNG exerts a more dominant protective effect. This study aimed to investigate the effects of ZNG on APAP‐induced liver damage, which, in addition to its therapeutic properties, also causes organ damage, through biochemical, molecular, and immunohistochemical analyses.

## 2 Materials and Methods

### Ethics Committee Approval

Ethical approval for the study was obtained from the Kafkas University Animal Experiments Local Ethics Committee (KAU‐HADYEK) (Ethical Approval No: KAU‐HADYEK/2025‐217), Kars, Türkiye. All animal experiments were carried out following the ARRIVE guidelines, the U.K. Animals (Scientific Procedures) Act, 1986, and associated guidelines, and EU Directive 2010 for animal experiments.

### 2.1 Rat Characteristics and Experimental Groups

In this study, a total of 28 male rats *Wistar Albino* weighing 220‐250 g were used. The animals were kept in cages in a controlled room with a constant 12‐h light‐dark cycle and temperature of 24°C–25°C. They were provided with unlimited access to water and standard food. In this study, with 7 animals in each group, 4 groups were formed.

**I‐Control:** On day 7, a single oral dose of saline was administered.

**II‐ZNG:** ZNG was administered at a dose of 50 mg/kg orally for 7 days [18].

**III‐APAP:** On day 7, a single dose of 3000 mg/kg orally was administered as APAP [19].

**IV‐APAP** + **ZNG:** ZNG was administered at a dose of 50 mg/kg orally for 7 days. On day 7, a single dose of APAP was administered at a dose of 3000 mg/kg orally.

At the end of the procedures, all experimental animals were sacrificed in accordance with ethical guidelines under intramuscular anesthesia with ketamine (35–50 mg/kg) and xylazine (5–10 mg/kg). Blood samples and liver tissue were then taken. Liver tissue was stored at −80°C for biochemical and molecular analysis. The remaining portion was preserved in 10% neutral buffered formaldehyde for histopathological examination.

### 2.2 Analysis of Liver Function Markers

Aspartate aminotransferase (AST) and alanine aminotransferase (ALT) activities were analyzed using commercial test kits (Ankara, Türkiye). Serum AST and ALT activities were measured using a Bio‐Tek microplate reader according to the instructions of the commercial kit (Winooski, VT, USA).

### 2.3 Analysis of GSH and Malondialdehyde (MDA) Levels in Liver Tissue

Liver tissue was homogenized in 1.15% potassium chloride buffer at a ratio of 1:10. A portion of the homogenate was centrifuged at 10,000 rpm for 20 min. GSH analysis was performed on the obtained supernatant. The remaining homogenate was centrifuged at 3500 rpm for 15 min. The resulting supernatant was used for MDA analysis. The method of Placer et al. [20] was used to analyze MDA levels. A pink color was formed as a result of the reaction between thiobarbituric acid (TBA) and MDA. The absorbance value of the pink color was measured at 532 nm using a NanoDrop (Bio‐Tek, Gen5, Epoch, USA) device. The method of Sedlak and Lindsay [21] was used to measure GSH levels. According to this analytical method, the yellow color is formed as a result of the reduction of 5,5‐Dithiobis(2‐nitrobenzoic) acid (DTNB) by sulfhydryl group compounds. The absorbance values of this color were determined by spectrophotometric measurement at 412 nm (Bio‐Tek, Gen5, Epoch, USA).

### 2.4 Analysis of Antioxidant Activities in Liver Tissue

Liver tissue was homogenized with 1.15% potassium chloride for catalase (CAT), superoxide dismutase (SOD), and glutathione peroxidase (GPx) activities, and the supernatant was obtained by centrifugation. The antioxidant status of liver tissue was determined by analyzing and evaluating GPx, SOD, and CAT activities. SOD activity was analyzed according to Sun et al. [22], CAT activity according to Aebi [23], and GPx activity according to Lawrence and Burk [24]. The absorbance of the resulting color was measured at a wavelength of 412 nm (Bio‐Tek, Gen5, Epoch, USA). The total protein content of liver tissue was determined according to the method of Lowry et al. [25]. SOD activity measured at a wavelength of 560 nm (Bio‐Tek, Gen5, Epoch, USA) is based on the reduction of nitrobluetetrazolium (NBT) by superoxide radicals produced by the xanthine‐xanthine oxidase system. When the enzyme is insufficient, maximum reduction occurs, and a dark blue color is observed. If sufficient SOD is present, the enzyme converts superoxide anion to hydrogen peroxide (H_2_O_2_), reducing NBT reduction and causing a color change. Formazan formation is inversely proportional to enzyme concentration. CAT measurement principle is based on the ability of H_2_O_2_ to absorb light at 240 nm (Bio‐Tek, Gen5, Epoch, USA).

### 2.5 RNA Extraction and Real‐Time Polymerase Chain Reaction (RT‐PCR) Analysis

According to the manufacturer's guidelines, total RNA was extracted from liver tissue using QIAzol Lysis Reagent (Qiagen, Germany). The concentration and purity of the RNA samples were evaluated using a NanoDrop® spectrophotometer (BioTek Epoch). cDNA was synthesized from 2 μg of total RNA using the High‐Capacity cDNA Kit (Applied Biosystems, Thermo Fisher Scientific, Lithuania). RT‐PCR was conducted using the Power SYBR Green Master Mix PCR kit (Qiagen, Germany) on the Rotor‐Gene Q 5plex HRM platform (Qiagen, Germany). The mRNA levels of nuclear factor erythroid 2‐related factor 2 (Nrf2), heme oxygenase‐1 (HO‐1) c‐Jun N‐terminal kinase (JNK), nuclear factor kappa B (NF‐κB), caspase‐3, tumor necrosis factor‐α (TNF‐α), activating transcription factor 6 (ATF‐6), interleukin 1β (IL‐1β), anti‐apoptotic B‐cell lymphoma 2 (Bcl‐2), Bcl‐2‐associated X protein (Bax), mitogen‐activated protein kinase 14 (MAPK14), glucose‐regulated protein 78 (GRP78), Beclin‐1, and light chain 3B (LC3B) in the liver tissues were analyzed in triplicate using gene‐specific primers (Table 1). β‐actin was used as a reference gene. The relative gene expression levels were determined from the Ct value and calculated using the 2^−ΔΔCt^ method, Livak and Schmittgen [26].

**Table 1:** Primer sequences of genes analyzed in real‐time polymerase chain reaction (PCR).

Gene	Accession Number	Primers	Product Size (bp)
β‐actin	NM_031144.3	F: GGAGATTACTGCCCTGGCTCCTAGCR: GGCCGGACTCATCGTACTCCTGCTT	155
Nrf2	NM_031789.3	F: TTTGTAGATGACCATGAGTCGCR: TCCTGCCAAACTTGCTCCAT	161
HO‐1	NM_012580.2	F: CACATCCGTGCAGAGAATTCTR: TTCGCTCTATCTCCTCTTCCAG	130
NF‐κB	NM_001415012.1	F: CCACTGTCAACAGCAGATGGR: TTTGCAGGCCCCACATAGTT	179
TNF‐α	NM_012675.3	F: ATGGGCTCCCTCTCATCAGT R: GCTTGGTGGTTTGCTACGAC	106
IL‐1β	NM_031512.2	F: GACTTCACCATGGAACCCGTR: GGAGACTGCCCATTCTCGAC	104
Bax	NM_017059.2	F: CCAGGACGCATCCACCAAGAAGCR: TGCCACACGGAAGAAGACCTCTCG	136
Bcl‐2	NM_016993.2	F: TATATGGCCCCAGCATGCGAR: GGGCAGGTTTGTCGACCTCA	136
Caspase‐3	NM_012922.3	F: GGAGCTTGGAACGCGAAGAAR: ACACAAGCCCATTTCAGGGT	169
ATF‐6	NM_001107196.1	F: CACAGACACGGATGATGTCCAGTTR: GTCTGAGCAGAAGTGGCTGCT	136
GRP78	NM_013083.2	F: CTGATTCCGAGGAACACTGTGGTGR: CTTTTGTCAGGGGTCGTTCACCT	118
JNK	NM_053829.2	F: GAATCAGACCCATGCTAAGCR: CCATGAGCTCCATGACTATG	149
MAPK14	NM_031020.3	F: GCACTACAACCAGACAGTGGATAR: CTCATGGCTTGGCATCCTGT	169
LC3B	NM_022867.2	F: GACATTACCACATGCACCACTGC R: CTGAAGCAGGCATGGACCAGAG	131
Beclin‐1	NM_001034117.1	F: GGCGGCTCCTATTCCATCAA R: TGAGGACACCCAAGCAAGAC	108

Abbreviations: ATF‐6, activating transcription factor 6; Bax, Bcl‐2‐associated X protein; Bcl‐2, anti‐apoptotic B‐cell lymphoma 2; GRP78, glucose‐regulated protein 78; HO‐1, heme oxygenase‐1; IL‐1β, interleukin 1β; JNK, c‐Jun N‐terminal kinase; LC3B, light chain 3B; MAPK14, mitogen‐activated protein kinase 14; NF‐κB, nuclear factor kappa B; Nrf2, nuclear factor erythroid 2‐related factor 2; TNF‐α, tumor necrosis factor‐α.

### 2.6 Immunohistochemical Analyses

Liver tissue sections taken from rats were subjected to xylene and alcohol washes and then rinsed with phosphate‐buffered saline (PBS). To inhibit endogenous peroxidase activity, the samples were incubated for 10 min in 3% H_2_O_2_ prepared in 0.1 M PBS. To reveal the antigens, the citrate buffer solution was heated to the maximum temperature in a microwave oven at 800 watts for 10 min. Blocking solution A was added to prevent non‐specific binding of Invitrogen Histostain Plus Broad Spectrum. Then, heat shock protein 60 (HSP60) (sc‐59567, diluted 1/100), heat shock protein 70 (HSP70) (sc‐32239, diluted 1/100), and heat shock protein 90 (HSP90) (sc‐13119, diluted 1/100) primary antibodies were applied to the sections and incubated overnight at +4°C. After incubation with the primary antibody, the Streptavidin‐biotin peroxidase technique, one of the indirect methods, was used. For secondary detection, an HRP system (Thermo Fisher, Cat. No: TP‐125‐HL) containing 3,3′‐Diaminobenzidine (DAB) as a chromogen and Gill's hematoxylin for counterstaining was used. To determine whether the immune reactivities were specific, all procedures were applied identically to sections incubated in PBS without the addition of primary antibodies. In the sections, the percentage of stained cells and the degree of staining were used as criteria for scoring using a semi‐quantitative method in the field. Immunohistochemical evaluations were performed by examining whether target cells stained, the character of the staining, and the staining intensity in the stained target cells. Immunostained preparations were evaluated using a scale of 0 to 3, indicating no staining (0), weak staining (+1), moderate staining (+2), and strong staining (+3). Cell staining intensity was assessed semi‐quantitatively from a total of 20 regions, with 5 regions randomly selected from each group and 4 areas defined within each region [27].

### 2.7 Statistical Analysis

Prior to statistical analysis, the conformity of the data to a normal distribution was assessed using the Shapiro‐Wilk test, and the homogeneity of variances was evaluated using Levene's test. For the biochemical parameters, which exhibited normal distribution and homogeneous variances, a one‐way analysis of variance (ANOVA) followed by Tukey's post hoc test was conducted to determine significant differences among the four groups. All biochemical analyses were performed using GraphPad® 10.1.0 (GraphPad Software, San Diego, CA, USA). Data obtained from immunohistochemical examinations (based on the staining degree of cells showing immunoreactivity) were analyzed using SPSS® 20 (IBM Corp., Armonk, NY, USA) software. Since the assumption of homogeneity of variances was violated for the immunohistochemical data, a one‐way ANOVA followed by Tamhane's post hoc test was utilized to compare differences between the groups. A *p*‐value less than 0.05 was considered statistically significant.

## 3 Results

### 3.1 Effects of APAP and ZNG Administration on Liver Function Markers

**Figure 1:**
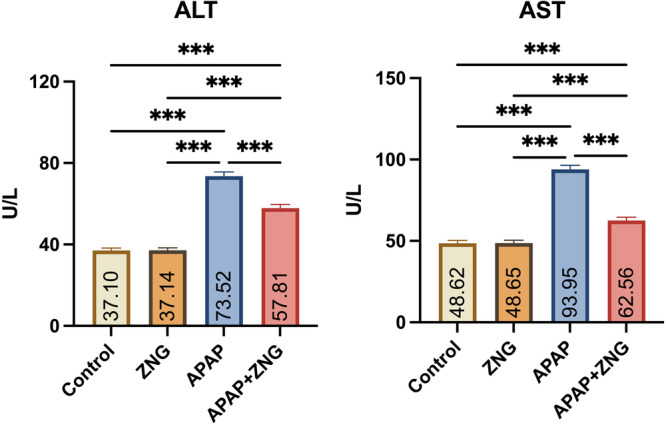
Means and SD of four groups for ALT and AST parameters. *p < 0.05, **p < 0.01, ***p < 0.001. ALT, Alanine aminotransferase; APAP, Acetaminophen; AST, Aspartate aminotransferase; ZNG, Zingerone.

Serum ALT and AST activities were analyzed to assess liver function (Figure 1). An increase was detected in the APAP group compared to the control (*p* < 0.001). When administered together with ZNG and APAP, it significantly reduced these increases (*p* < 0.001). No statistically significant difference was observed between the control and ZNG groups (*p* > 0.05).

### 3.2 Effects of APAP and ZNG Administration on Oxidative Stress Parameters

**Figure 2:**
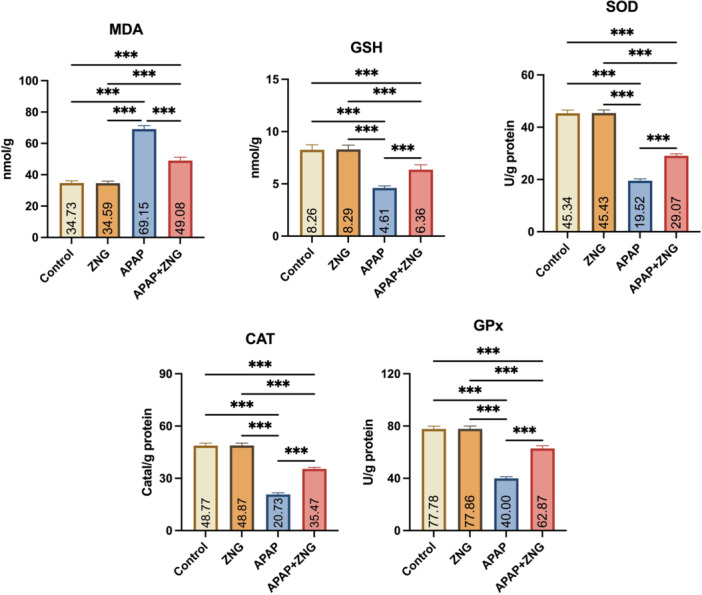
Means and SD of four groups for oxidative stress parameters. *p < 0.05, **p < 0.01, ***p < 0.001. APAP, acetaminophen; CAT, catalase; GPx, glutathione peroxidase; GSH, glutathione; MDA, malondialdehyde; SOD, superoxide dismutase; ZNG, zingerone.

In the APAP group, antioxidant markers (GSH, CAT, GPx, SOD) were decreased (*p* < 0.001), while MDA levels were increased (*p* < 0.001) compared to the control group. ZNG treatment effectively reversed these changes, increasing antioxidants while decreasing MDA levels (*p* < 0.001). No statistically significant difference was observed between the control and ZNG groups (*p* > 0.05) (Figure 2).

### 3.3 Effects of APAP and ZNG Administration on Nrf2 and HO‐1 Markers

**Figure 3:**
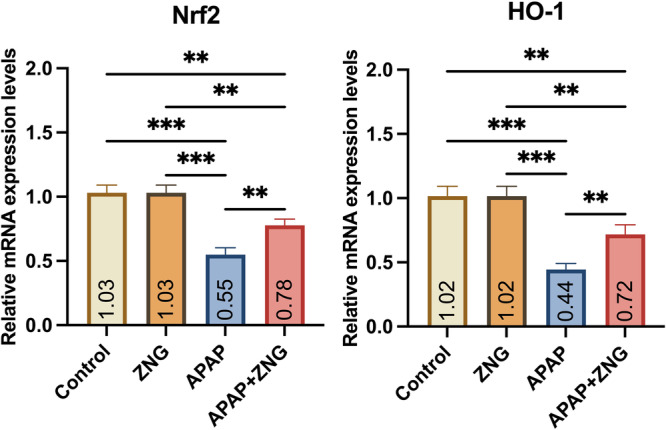
Means and SD of four groups for Nrf2 and HO‐1 parameters. *p < 0.05, **p < 0.01, ***p < 0.001. APAP, acetaminophen; HO‐1, heme oxygenase‐1; Nrf2, nuclear factor erythroid 2‐related factor 2; ZNG, zingerone.

Expression levels of Nrf2 and HO‐1 genes in liver tissue were analyzed using RT‐PCR. Only in the APAP group were the expression levels of these genes significantly reduced compared to the control group (Figure 3). In contrast, in the group treated with ZNG concurrently with APAP, the expression levels of these genes were significantly increased compared to the APAP‐only group.

### 3.4 Effects of APAP and ZNG Administration on Inflammatory Markers

**Figure 4:**
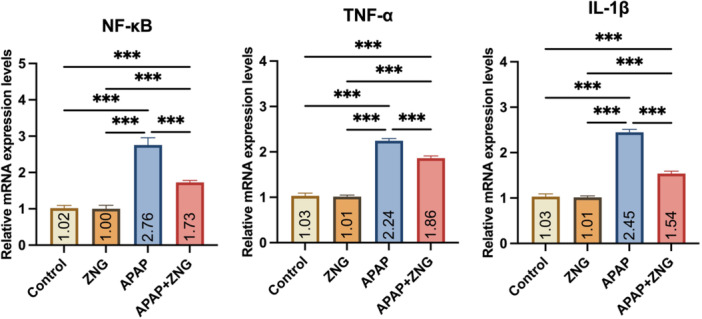
Means and SD of four groups for inflammation parameters. *p < 0.05, **p < 0.01, ***p < 0.001. APAP, acetaminophen; IL‐1β, interleukin 1β; NF‐κB, nuclear factor kappa B; TNF‐α, tumor necrosis factor‐α; ZNG, zingerone.

APAP exposure increased NF‐κB, TNF‐α, and IL‐1β mRNA transcription levels compared to the control group (*p* < 0.001). ZNG treatment suppressed this effect of APAP and reduced NF‐κB, TNF‐α, and IL‐1β (*p* < 0.001) mRNA transcription levels. No statistically significant difference was observed between the control and ZNG groups (*p* > 0.05) (Figure 4).

### 3.5 Effects of APAP and ZNG Administration on Apoptotic Markers

**Figure 5:**
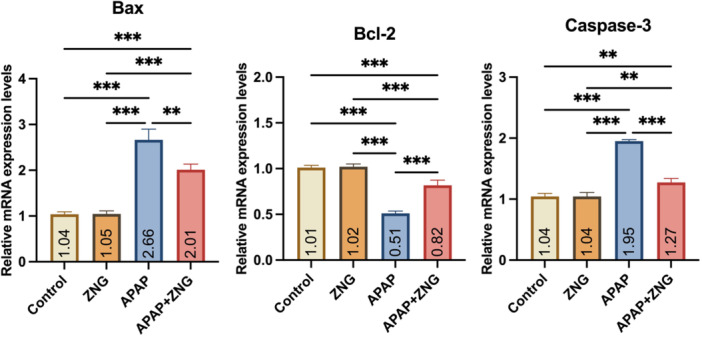
Means and SD of four groups for apoptosis parameters. *p < 0.05, **p < 0.01, ***p < 0.001. APAP, acetaminophen; Bax, Bcl‐2‐associated X protein; Bcl‐2, anti‐apoptotic B‐cell lymphoma 2; ZNG, zingerone.

In the APAP group, a significant increase in Bax and caspase‐3 parameters and a decrease in Bcl‐2 were observed compared to the control group (*p* < 0.001). ZNG treatment reversed these changes, exhibiting antiapoptotic properties. No statistically significant difference was observed between the control and ZNG groups (*p* > 0.05) (Figure 5).

### 3.6 Effects of APAP and ZNG Administration on Endoplasmic Reticulum Stress Markers

**Figure 6:**
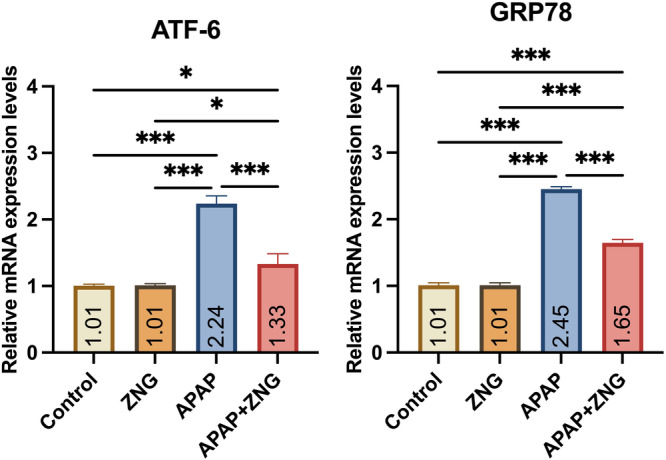
Means and SD of four groups for endoplasmic reticulum stress parameters. *p < 0.05, **p < 0.01, ***p < 0.001. APAP, acetaminophen; ATF‐6, activating transcription factor 6; GRP78, glucose‐regulated protein 78; ZNG, zingerone.

Compared to the control group, the expression of endoplasmic reticulum stress markers (ATF‐6 and GRP78) was significantly higher in the APAP group (*p* < 0.001). When ZNG was administered concomitantly with APAP, decreases in ATF‐6 and GRP78 mRNA transcription levels were observed (*p* < 0.001). No statistically significant difference was observed between the control and ZNG groups (*p* > 0.05) (Figure 6).

### 3.7 Effects of APAP and ZNG Administration on JNK and MAPK14 Parameters

**Figure 7:**
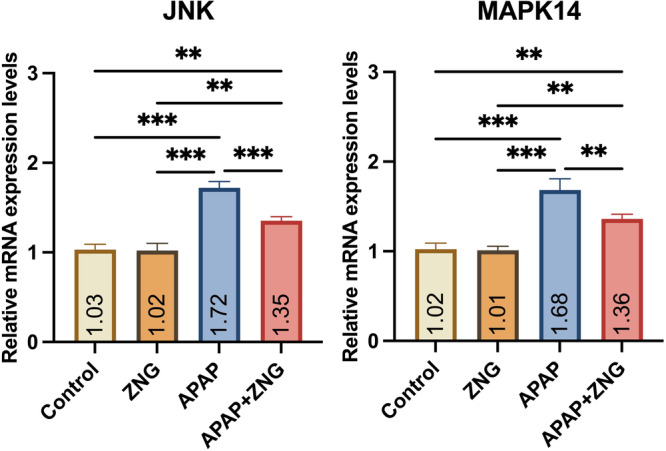
Means and SD of the four groups for JNK and MAPK14 parameters. *p < 0.05, **p < 0.01, ***p < 0.001. APAP, acetaminophen; JNK, c‐Jun N‐terminal kinase; MAPK14, mitogen‐activated protein kinase 14; ZNG, zingerone.

The analysis results for the JNK and MAPK14 parameters are shown in Figure 7. The study results showed that JNK and MAPK14 analysis demonstrated a statistically significant increase in the APAP group compared to the control group (*p* < 0.001). Furthermore, a statistically significant difference was found between the APAP and APAP + ZNG groups in the JNK parameter (*p* < 0.01) and the MAPK14 parameter (*p* < 0.01). No statistically significant difference was observed between the control and ZNG groups (*p* > 0.05).

### 3.8 Effects of APAP and ZNG Administration on Autophagy Markers

**Figure 8:**
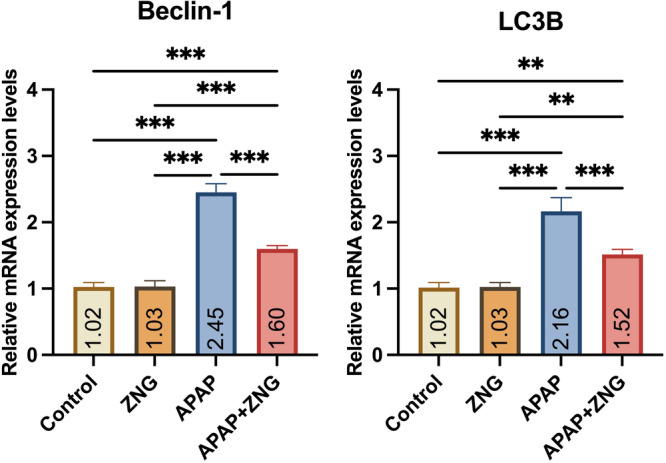
Means and SD of four groups for autophagy parameters. *p < 0.05, **p < 0.01, ***p < 0.001. APAP, Acetaminophen; LC3B, Light chain 3B; ZNG, Zingerone.

The analysis results for the Beclin‐1 and LC3B parameters are shown in Figure 8. The study results showed that Beclin‐1 and LC3B analysis demonstrated a statistically significant increase in the APAP group compared to the control group (*p* < 0.001). Furthermore, statistically significant differences were found between the APAP and APAP + ZNG groups in the Beclin‐1 parameter (*p* < 0.001) and the LC3B parameter (*p* < 0.01). No statistically significant difference was observed between the control and ZNG groups (*p* > 0.05).

### 3.9 Effects of APAP and ZNG Administration on HSP60, HSP70, and HSP90

**Figure 9:**
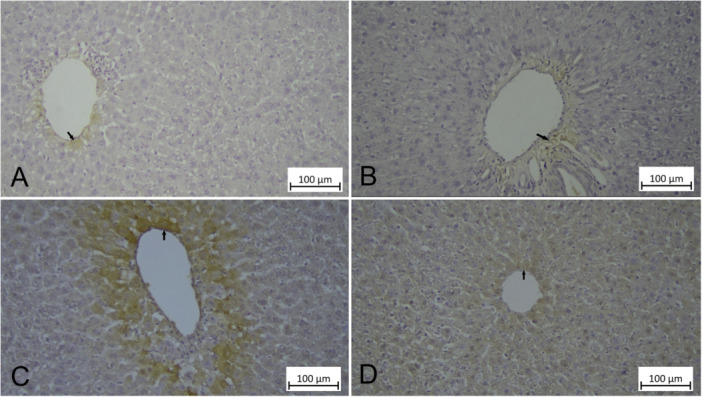
Rat liver tissue HSP60 immunoreactivity. (A) Control, arrow: weak immunopositivity; (B) ZNG, arrow: weak immunopositivity; (C) APAP, arrow: moderate immunopositivity; D) APAP + ZNG, arrow: weak immunopositivity. IHC staining. APAP, Acetaminophen; HSP60, Heat shock protein 60; ZNG, Zingerone.

**Figure 10:**
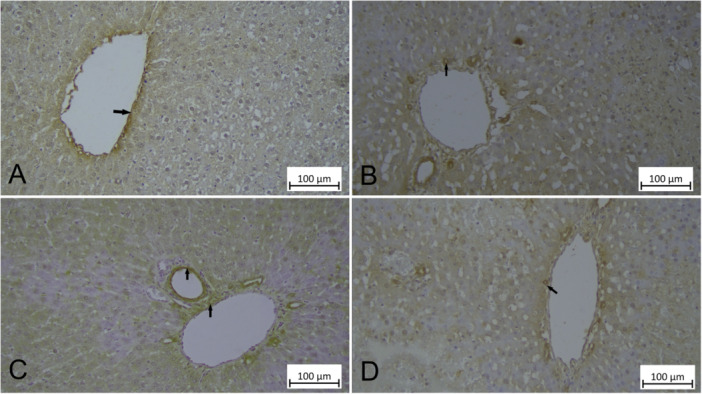
Rat liver tissue HSP70 immunoreactivity. (A) Control, arrow: moderate immunopositivity; (B) ZNG, arrow: moderate immunopositivity; (C) APAP, arrow: strong immunopositivity; (D) APAP + ZNG, arrow: moderate immunopositivity. IHC staining. APAP, Acetaminophen; HSP70: Heat shock protein 70; ZNG, Zingerone.

**Figure 11:**
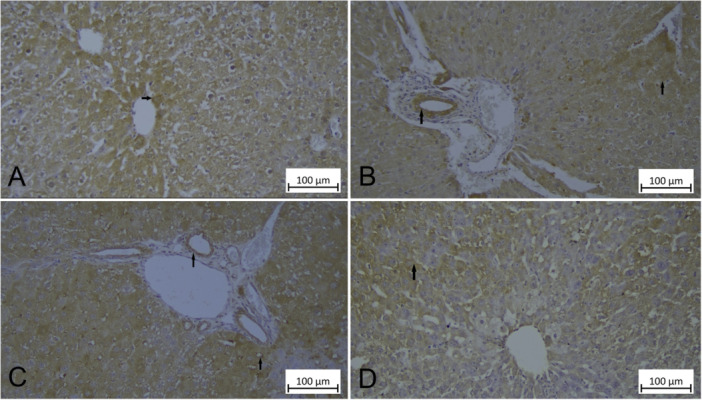
Rat liver tissue HSP90 immunoreactivity. (A) Control, arrow: moderate immunopositivity; (B) ZNG, arrow: moderate immunopositivity; (C) APAP, arrow: strong immunopositivity; (D) APAP + ZNG, arrow: moderate immunopositivity. IHC staining. APAP, Acetaminophen; HSP90, Heat shock protein 90; ZNG, Zingerone.

Statistical analysis performed to compare HSP60 immunoreactivity values according to groups revealed weak (+1) HSP60 immunoreactivity in hepatocytes around the central vein and epithelial cells lining the ductus bilifera wall in liver tissue of rats in the control (Figure 9A), ZNG (Figure 9B), and APAP + ZNG (Figure 9D) groups. In rats in the APAP group (Figure 9C), moderate (+2) HSP60 immunoreactivity was observed in hepatocytes surrounding the central vein and in epithelial cells lining the wall of the ductus bilifera in the liver tissue. Statistical analysis performed to compare HSP70 and HSP90 immunoreactivity values revealed moderate (+2) HSP70 and HSP90 immunoreactivity in hepatocytes surrounding the central vein and epithelial cells lining the ductus bilifera wall in the control (Figures 10A, 11A), ZNG (Figures 10B, 11B), and APAP + ZNG (Figures 10D, 11D) groups. In the APAP group (Figures 10C, 11C), strong (+3) HSP70 and HSP90 immunoreactivity was observed in hepatocytes surrounding the central vein and in epithelial cells lining the wall of the ductus bilifera in liver tissue of rats (Table 2).

**Table 2:** Semiquantitative analysis of HSP60, HSP70, and HSP90 immunoreactivity in liver tissue.

Groups	HSP60	HSP70	HSP90
Control	0.96 ± 0.36^b^	1.95 ± 0.56^b^	2.30 ± 0.57^b^
ZNG	0.91 ± 0.45^b^	1.98 ± 0.53^b^	2.38 ± 0.51^b^
APAP	1.93 ± 0.4^a^	2.73 ± 0.44^a^	2.73 ± 0.38^a^
APAP + ZNG	1.00 ± 0.36^b^	1.76 ± 0.32^b^	2.00 ± 0.32^b^
F	29.86	16.31	8.50
*P* value	0.001	0.001	0.001

* Different letters (a, b) in each column represent statistically significant differences. APAP, acetaminophen; HSP60, heat shock protein 60; HSP70, heat shock protein 70; HSP90, heat shock protein 90; ZNG, zingerone.

## 4 Discussion

Acetaminophen, used as an antipyretic and analgesic, is known to cause various tissue damages. This damage is associated with the activation of damage pathways caused by oxidative stress. Therefore, this study investigated the effects of ZNG, a natural antioxidant, on APAP‐induced liver damage.

Transaminase enzymes are frequently used as markers of liver damage. In particular, AST and ALT activities are biomarkers of hepatic damage [1, 28]. Hepatic damage increases membrane permeability, allowing enzymes to pass into the bloodstream [1, 5]. AST and ALT are markers of liver damage because they leak from the cells into the serum circulation when hepatocyte integrity is compromised [1]. Additionally, they catalyze the transfer of amino groups in amino acid metabolism and gluconeogenesis. Although AST and ALT are found in high concentrations in the liver, they can also be found in other tissues [29]. Analysis of AST and ALT enzymes is helpful in predicting APAP damage [30]. This study found that APAP administration increased AST and ALT activities in the liver tissue of rats, resulting in liver tissue damage. Studies on this subject have reported that the reason APAP application increases liver enzymes may be due to the destruction and severe damage of hepatocytes in the liver [31, 32]. Both APAP and ZNG administration were found to reduce increased AST and ALT activities in the liver. The fact that APAP + ZNG therapy reduces increased AST and ALT activities in the liver suggests that these treatments may have protective effects on hepatocyte integrity. ZNG has been shown to reduce liver cell death in response to various toxic substances, thereby preventing liver enzymes from leaking into the bloodstream [33, 34].

Oxidative stress, characterized by the production and accumulation of reactive oxygen species in tissues and cells, results from an imbalance in the detoxification of these reactive oxygen species [7]. Antioxidants present in the organism form a defense line against the oxidative stress that occurs [35, 36]. Molecular damage occurs due to impaired signal transduction resulting from a decrease in the antioxidant defense line and increased oxidant production [9, 37]. Oxidative stress, associated with hepatotoxicity, damages cellular structures such as nucleic acids, lipids, and proteins [9, 36]. Oxidative stress plays a major role in the pathogenesis of APAP‐induced liver damage [7, 19]. Nrf2 is a transcription factor that promotes transcription by binding to antioxidant response elements. Activation of Nrf2 increases the production of antioxidant molecules and reduces oxidative stress [38]. Furthermore, protective genes such as Nrf2 are activated to protect against apoptosis caused by reactive oxygen species [39]. Nrf2 induces the expression of HO‐1. This enzyme exhibits cytoprotective, antioxidant, and anti‐inflammatory effects. HO‐1, also called heat shock protein‐32, is the inducible isoform of HO that catalyzes the breakdown of heme into biliverdin, carbon monoxide, and free iron. HO‐1 is an important phase II and anti‐inflammatory enzyme that is upregulated in oxidative stress via Nrf2 [40]. In the present study, it was found that in rat liver tissue damage caused by APAP, MDA levels increased, GSH and Nrf2 levels decreased, and CAT, GPx, HO‐1, and SOD enzyme activities decreased. As a result, oxidative stress was found to occur, and this oxidative stress caused damage to liver tissue. Studies have shown that APAP triggers oxidative stress by depleting GSH stores and increasing lipid peroxidation, and that the resulting oxidative stress impairs liver tissue integrity [41, 42]. In the presented study, both APAP and ZNG treatments were found to decrease MDA levels, increase decreased GSH and Nrf2 levels, and increase CAT, GPx, HO‐1, and SOD activities. In APAP‐induced damage, excessive reactive metabolites deplete cellular GSH stores, leading to increased oxidative stress. Therefore, the decrease in MDA levels and the increase in GSH, CAT, GPx, and SOD activities suggest that the applied treatment mitigates APAP‐induced oxidative damage and contributes to the restoration of redox homeostasis in liver tissue. ZNG has been reported to suppress oxidative stress by reducing lipid peroxidation caused by various toxic substances, and this suppression of oxidative stress is attributed to its antioxidant properties in liver tissue [33, 34].

Oxidative stress caused by APAP triggers a range of inflammatory responses [43]. In APAP‐induced liver damage, NF‐κB, which is associated with oxidative stress, triggers the active mediator, initiating the transcription of inflammatory mediators [43, 44]. Increased NF‐κB levels resulting from inflammation increase the levels of pro‐inflammatory cytokines such as IL‐1β and TNF‐α produced by macrophages/monocytes [44, 45]. Increased IL‐1β and TNF‐α activate neutrophils and monocytes, thereby enhancing the inflammatory response [43]. Inflammation caused by APAP pathologically damages the structure of the liver [27]. Reducing the production of pro‐inflammatory cytokines reduces APAP‐induced liver damage [46]. In the current study, it was found that APAP increased the expression levels of NF‐κB, IL‐1β, and TNF‐α in the liver tissue of rats, causing inflammation, and that this inflammation led to damage in the liver tissue. Studies on this subject have indicated that in rats treated with APAP, oxidative stress in the liver tissue leads to an increase in oxidants, disrupting the integrity of cell membranes and triggering inflammation through increased free radicals [47, 48]. In the presented study, APAP + ZNG treatment was found to reduce increased NF‐κB, IL‐1β, and TNF‐α mRNA expression levels. NF‐κB is a central regulator that acts as a bridge between oxidative stress and inflammation. Therefore, when both the improvement in antioxidant parameters and the decrease in pro‐inflammatory gene expression are considered together in the APAP + ZNG group, it is thought that the protective effect of the treatment is due not only to free radical scavenging but also to the suppression of NF‐κB‐mediated inflammatory signaling pathways. This may be one of the key mechanisms underlying the observed hepatoprotective effect. Studies have shown that ZNG, a natural compound, suppresses oxidative stress by preventing lipid peroxidation due to its antioxidant effect against toxic substances, and as a result of suppressing oxidative stress, it prevents inflammation with its anti‐inflammatory effect and exhibits hepatoprotective properties [18, 49].

Intracellular and extracellular stimuli such as oxidative stress, cytokines, and endoplasmic reticulum stress activate MAPK [50, 51]. The sub‐member of MAPK is the JNK signaling pathway [51]. These signaling pathways regulate various cellular activities such as survival, death, differentiation, and proliferation [50]. MAPK, which is associated with cell death, is responsible for the production of reactive oxygen species and cytokines [52, 53]. Continuous activation of JNK leads to hepatocyte death through increased mitochondrial permeability, translocation into mitochondria, and increased mitochondrial reactive oxygen species. It also triggers apoptosis by promoting cell proliferation [54]. The JNK/MAPK signaling pathway plays a critical role in APAP‐induced liver damage [51, 53]. In the presented study, it was determined that RT‐PCR results increased the levels of APAP, MAPK14, and JNK in liver tissue. Studies have shown that APAP causes apoptosis by activating the JNK/MAPK signaling pathway in the liver [53, 55]. In the current study, both APAP and ZNG treatments were found to reduce the activation of the JNK/MAPK signaling pathway in the liver, which is increased by APAP administration. The JNK/MAPK suppression observed with APAP and ZNG applications may indicate not only a signaling change but also a disruption of the oxidative stress–mitochondrial damage–inflammation axis. Studies have shown that naturally occurring compounds with antioxidant properties prevent APAP damage by blocking JNK/MAPK phosphorylation [56, 57].

Apoptotic cascades are associated with liver damage caused by APAP [58]. Increased reactive oxygen species cause mitochondrial damage, activating the caspase cascade and leading to apoptosis [43, 59]. Apoptosis is the most important mechanism of APAP‐induced hepatocyte death through the depletion of antiapoptotic proteins and the overexpression of proapoptotic proteins [58]. The death ligand TNF‐α binds to specific death receptors, leading to the activation and degradation of caspase‐3, and ultimately to apoptosis [46, 60]. Caspase‐3, involved in the apoptosis cascade of liver cells, is upregulated in APAP‐induced damage [60]. The ratio of the proapoptotic protein Bax to the antiapoptotic protein Bcl‐2 plays a significant role in hepatocyte apoptosis [43, 46]. In the present study, RT‐PCR analysis of APAP revealed that it increased Bax and caspase‐3 expression and decreased Bcl‐2 expression. Studies on this subject have indicated that APAP application affects apoptosis activation by increasing reactive oxygen species in liver tissue and that it triggers caspase‐3 activation by increasing oxidative stress markers such as hydrogen peroxide, leading to apoptosis in liver tissue [42, 61]. In the presented study, both APAP and ZNG treatments were found to decrease Bax and caspase‐3 expression and increase Bcl‐2 expression in rat liver tissue, as determined by RT‐PCR analysis. In APAP‐induced liver damage, excessive oxidative stress and inflammation trigger mitochondrial damage, altering the Bax/Bcl‐2 balance in a proapoptotic direction and leading to caspase‐dependent cell death. The decrease in Bax and caspase‐3 expression and the increase in Bcl‐2 expression observed in our study suggest that the treatments suppress the mitochondrial apoptotic pathway. Various studies have reported that ZNG reduces increased reactive oxygen species in liver tissue due to its strong antioxidant properties against toxic substances, regulates inflammation parameters due to its strong anti‐inflammatory effect, and improves apoptosis in liver tissue due to its antiapoptotic properties [33, 62].

Endoplasmic reticulum stress, a conserved intracellular signaling network, is a crucial intrinsic stressor that supports cellular homeostasis and survival [63]. Drug‐induced liver damage is associated with endoplasmic reticulum stress. Suppression of endoplasmic reticulum stress can prevent liver damage [64]. The unfolded protein response to endoplasmic reticulum stress is an adaptive response and plays an important role in the pathogenesis of liver diseases [65]. In addition, endoplasmic reticulum stress plays an important role in the pathogenesis of APAP‐induced liver damage [66]. Markers such as ATF‐6, an endoplasmic reticulum stress marker, regulate the inhibition of protein synthesis and the upregulation of molecular chaperones [65]. In the present study, it was found that APAP application increased the mRNA expression levels of GRP78 and ATF‐6, which are endoplasmic reticulum stress markers. Studies have shown that APAP induces endoplasmic reticulum stress through the induction of multiple signaling pathways such as oxidative stress and inflammation, and that consequently, endoplasmic reticulum stress may play a role in the pathophysiology of APAP‐induced liver damage [64, 67]. In the presented study, it was found that APAP + ZNG treatment reduced the expression levels of endoplasmic reticulum stress markers GRP78 and ATF‐6, which were increased by APAP administration. The hepatoprotective effect of APAP + ZNG is thought to occur through the regulation of the oxidative stress‐endoplasmic reticulum stress‐inflammation‐apoptosis axis. A study reported that ZNG treatment reduced endoplasmic reticulum stress markers, and this reduction was attributed to a decrease in unfolded protein response, which in turn was linked to a decrease in apoptotic marker levels [14].

Autophagy, a programmed intracellular degradation process common in eukaryotic cells that can eliminate dysfunctional organelles and proteins, plays a role in cell division and growth [68, 69]. If autophagy occurs, it leads to cellular dysfunction [70, 71]. Beclin‐1 is an important biomarker in autophagic pathway tracking and autophagosome formation [70]. Abnormal autophagy is associated with the development of diseases [68]. When the antioxidant and oxidation balance of an organism is disrupted, biomolecules undergo oxidative damage [72]. Oxidative stress triggers apoptosis, and there is an interaction between the resulting apoptosis and autophagy [69, 73]. Apoptosis and autophagy lead to cell death, after which damage occurs in tissues and organs [69, 74]. Autophagy is not only triggered during apoptosis but also increases in conditions such as endoplasmic reticulum stress [75]. Autophagy plays an important role in the pathological processes of liver damage [76]. In the presented study, it was determined that APAP application increased the expression levels of Beclin‐1 and LC3B in liver tissue according to RT‐PCR results. As a result, autophagy occurs in the liver tissue. Studies have shown that APAP application increases reactive oxygen species, that these increased reactive oxygen species trigger oxidative stress, leading to apoptosis, and that the resulting apoptosis triggers autophagy activation [77, 78]. In the current study, APAP + ZNG treatment was found to reduce the expression levels of Beclin‐1 and LC3B in liver tissue according to RT‐PCR results. The decrease in these markers after APAP + ZNG treatment suggests that the need for autophagic activation is reduced due to the alleviation of cellular stress. This treatment was found to improve autophagy in liver tissue. Studies have shown that natural compounds reduce reactive oxygen species in toxic substance damage, and this reduction in reactive oxygen species improves autophagy [79, 80].

Various members of the heat shock protein family are molecular chaperones and resist the denaturation of other stress‐induced proteins [81]. Heat shock proteins are found in all organisms and play a role in repair and cell protection under stress conditions [82]. HSP60, HSP70, and HSP90 are the main heat shock proteins in response to cellular stress. HSP60 and HSP70, in particular, are used as indicators of oxidative stress [81]. They are considered biomarkers in the assessment of stress in different species [79]. Their expression increases under adverse conditions, while they are found at low levels in normal physiological conditions [83]. In the current study, HSP60 immunoreactivity was found to be weak in the control, ZNG, and APAP + ZNG groups, and moderate in the APAP group; HSP70 and HSP90 immunoreactivity were found to be moderate in the control, ZNG, and APAP + ZNG groups, and strong in the APAP group. The fact that these proteins are at levels close to control levels in the APAP + ZNG group suggests that the treatment reduces mitochondrial damage and protein folding stress and maintains cellular homeostasis. Studies have shown that while toxic substances increase the levels of heat shock proteins by triggering lipid peroxidation, natural antioxidant compounds inhibit the expression of heat shock proteins and improve cell viability by stimulating antioxidant enzyme levels and reducing reactive oxygen species [83, 84].

In this study, ZNG was administered orally for 7 days, and APAP was administered orally as a single dose on the last day of the study. Therefore, the study is of a “pre‐treatment/prophylactic design”.

## 5 Conclusion

When the findings are evaluated together, it is seen that APAP administration disrupts the normal structure of the liver by triggering oxidative stress, inflammation, heat shock proteins, endoplasmic reticulum stress, autophagy, and apoptosis, while ZNG administration effectively mitigates these cellular changes in this experimental model. These preclinical findings indicate that ZNG has protective mechanisms against APAP‐induced liver damage. However, further research and clinical trials are needed before ZNG can be evaluated as a therapeutic agent.

## Author Contributions

**Serpil Aygörmez:** writing – original draft, writing – review and editing, visualization, supervision, resources, methedology, investigation, formal analysis, data curation, conceptualization. **Mustafa Makav:** writing – review and editing, visualization, supervision, resources, investigation, methodology, formal analysis, conceptualization, data curation. **Sevda Eliş Yıldız:** resources, methodology, investigation, formal analysis, data curation, conceptualization. **Elif Dalkılınç:** validation, supervision, resources, methodology, methodology, investigation, formal analysis, data curation, conceptualization. **Mushap Kuru:** validation, supervision, resources, methodology, investigation, formal analysis, conceptualization, data curation. **Şaban Maraşlı:** supervision, resources, methodology, investigation, formal analysis, data curation, conceptualization.

## Funding

The authors have nothing to report.

## Conflicts of Interest

The authors declare no conflicts of interest.

## Data Availability

The data that support the findings of this study are available from the corresponding author upon reasonable request.

## References

[1] M. T. Islam , C. Quispe , M. A. Islam , et al., “Effects of Nerol on Paracetamol‐Induced Liver Damage in Wistar Albino Rats,” Biomedicine and Pharmacotherapy 140 (2021): 111732. 34130201 10.1016/j.biopha.2021.111732

[2] M. T. Islam , M. M. Medha , M. A. Islam , et al., “Protective Effects of Sclareol Against Paracetamol‐Induced Liver Damage in Rats, Possibly Through an Oxidative Stress‐Reducing Pathway,” Pharmacological Research – Natural Products 6 (2025): 100132.

[3] H. A. E. Fadil , A. Behairy , L. L. Ebraheim , Y. M. Abd‐Elhakim , and H. H. Fathy , “The Palliative Effect of Mulberry Leaf and Olive Leaf Ethanolic Extracts on Hepatic CYP2E1 and Caspase‐3 Immunoexpression and Oxidative Damage Induced by Paracetamol in Male Rats,” Environmental Science and Pollution Research International 30, no. 14 (2023): 41682–41699. 36637651 10.1007/s11356-023-25152-z PMC10067661

[4] K. D. Ayenew and Y. Wasihun , “Hepatoprotective Effect of Methanol Extract of Agave Americana Leaves on Paracetamol Induced Hepatotoxicity in Wistar Albino Rats,” BMC Complementary Medicine and Therapies 23, no. 1 (2023): 99. 37005601 10.1186/s12906-023-03931-y PMC10067186

[5] M. F. Koshak , M. Z. El‐Readi , M. E. Elzubier , et al., “Antioxidative and Anti‐Inflammatory Protective Effects of Fucoxanthin Against Paracetamol‐Induced Hepatotoxicity in Rats,” Marine Drugs 21, no. 11 (2023): 592. 37999416 10.3390/md21110592 PMC10672227

[6] C. Sarkar , M. Mondal , K. Al‐Khafaji , et al., “GC–MS Analysis, and Evaluation of Protective Effect of Piper Chaba Stem Bark Against Paracetamol‐Induced Liver Damage in Sprague‐Dawley Rats: Possible Defensive Mechanism by Targeting CYP2E1 Enzyme Through in Silico Study,” Life Sciences 309 (2022): 121044. 36208657 10.1016/j.lfs.2022.121044

[7] A. A. E. Latif , D. H. Assar , E. M. Elkaw , et al., “Protective Role of *Chlorellavulgaris* With Thiamine Against Paracetamol Induced Toxic Effects on Haematological, Biochemical, Oxidative Stress Parameters and Histopathological Changes in Wistar Rats,” Scientific Reports 11, no. 1 (2021): 3911. 33594164 10.1038/s41598-021-83316-8 PMC7887200

[8] C. D. Teixeira , P. O. Barbosa , W. G. Lima , et al., “Preventive Treatment With Guarana Powder (*Paulliniacupana*) Mitigates Acute Paracetamol‐Induced Hepatotoxicity by Modulating Oxidative Stress,” Toxicology Reports 14 (2025): 101946. 39989980 10.1016/j.toxrep.2025.101946 PMC11847530

[9] G. I. Edo , F. O. Onoharigho , A. N. Jikah , and J. J. Agbo , “The Ameliorative Effect of Methanol Extract of *Ricinodendronheudelotii* (Baill.) Leaves on Paracetamol‐Induced Hepatotoxicity in Wistar Rats,” Drug and Chemical Toxicology 48, no. 1 (2025): 98–106. 38839563 10.1080/01480545.2024.2362891

[10] E. K. M. Khedre , A. M. Hegab , A. A. El‐Mahis , et al., “Ziziphus Spina‐Christi Alleviates Paracetamol‐Induced Hepatorenal Toxicity in Rats Through In Vivo and Computational Approaches,” Scientific Reports 15, no. 1 (2025): 30163. 40825988 10.1038/s41598-025-14454-6 PMC12361524

[11] H. Şimşek , S. Küçükler , C. Gür , M. İleritürk , S. Aygörmez , and F. M. Kandemir , “Protective Effects of Zingerone Against Sodium Arsenite‐Induced Lung Toxicity: A Multi‐Biomarker Approach,” Iranian Journal of Basic Medical Sciences 26, no. 9 (2023): 1098–1106. 37605724 10.22038/IJBMS.2023.71905.15623 PMC10440130

[12] A. Dutta , G. Gurusubramanian , and V. K. Roy , “Zingerone Supplementation Affects Proliferation, Apoptosis, Antioxidant, and GLUT4 and Insulin Receptor Expression in Uterus of Mice,” Journal of Steroid Biochemistry and Molecular Biology 255, no. 2025: 106885. 41176169 10.1016/j.jsbmb.2025.106885

[13] A. Oviosun , E. C. Oviosun , N. J. Nto , A. E. Memudu , and E. G. Anyanwu , “Zingerone Attenuates Cadmium‐Induced Neuroinflammation, Oxidative Stress and Cognitive Deficit on the Prefrontal Cortex of Adult Wistar Rats,” Journal of Experimental Pharmacology 13 (2025): 323–341. 10.2147/JEP.S519571 PMC12174926 40535155

[14] S. Ç. Tuncer , C. Gur , S. Kucukler , S. A. Akarsu , and F. M. Kandemir , “Effects of Zingerone on Rat Induced Testicular Toxicity by Sodium Arsenite via Oxidative Stress, Endoplasmic Reticulum Stress, Inflammation, Apoptosis, and Autophagy Pathways,” Iranian Journal of Basic Medical Sciences 27, no. 5 (2024): 603610. 10.22038/IJBMS.2024.73342.15934 PMC11017849 38629098

[15] S. Aygörmez , M. Makav , M. Kuru , et al., “Biochemical and Immunohistochemical Evaluation of the Effects of Zingerone Against Colistin‐Induced Lung Injury in Ovariectomized Rats,” Journal of Biochemical Molecular Toxicology 39, no. 10 (2025): e70561. 41071670 10.1002/jbt.70561

[16] F. K. Ornek , G. C. Dincel , F. Basak , et al., “Zingerone as a Neuroprotective Agent in Experimental Diabetes: Evidence From Oxidative Stress, Inflammatory, and Apoptotic Markers,” Naunyn‐Schmiedeberg's Archives of Pharmacology Early View (2026). 10.1007/s00210-026-05268-y PMC13357459 41989597

[17] S. M. Ismail , Z. E. Eldin , M. H. Elbakkay , et al., “Enhancement of the Anti‐Inflammatory Effect of Zingerone Using the Galactosylated Chitosan Nano Formula Against CCl4‐Induced Hepatic Fibrosis: An In Vivo Study,” Journal of Pharmaceutical Innovation 21, no. 4 (2026): 419.

[18] S. Kucukler , E. Darendelioğlu , C. Caglayan , A. Ayna , S. Yıldırım , and F. M. Kandemir , “Zingerone Attenuates Vancomycin‐Induced Hepatotoxicity in Rats Through Regulation of Oxidative Stress, Inflammation and Apoptosis,” Life Sciences 259 (2020): 118382. 32898532 10.1016/j.lfs.2020.118382

[19] I. T. Henneh , W. Ahlidja , J. Alake , et al., “ *Ziziphusabyssinica* Root Bark Extract Ameliorates Paracetamol‐Induced Liver Toxicity in Rats Possibly via the Attenuation of Oxidative Stress,” Toxicology Reports 9 (2022): 1929–1937. 36518453 10.1016/j.toxrep.2022.10.012 PMC9743660

[20] Z. A. Placer , L. L. Cushman , and B. C. Johnson , “Estimation of Product of Lipid Peroxidation (Malonyl Dialdehyde) in Biochemical Systems,” Analytical Biochemistry 16, no. 2 (1966): 359–364. 6007581 10.1016/0003-2697(66)90167-9

[21] J. Sedlak and R. H. Lindsay , “Estimation of Total, Protein‐Bound, and Nonprotein Sulfhydryl Groups in Tissue With Ellman's Reagent,” Analytical Biochemistry 25 (1968): 192–205. 4973948 10.1016/0003-2697(68)90092-4

[22] Y. I. Sun , L. W. Oberley , and Y. Li , “A Simple Method for Clinical Assay of Superoxide Dismutase,” Clinical Chemistry 34, no. 3 (1988): 497–500. 3349599

[23] H. Aebi , “[13] Catalase in Vitro,” Methods in Enzymology 105 (1984): 121–126. 6727660 10.1016/s0076-6879(84)05016-3

[24] R. A. Lawrence and R. F. Burk , “Glutathione Peroxidase Activity in Selenium‐Deficient Rat Liver,” Biochemical and Biophysical Research Communications 71, no. 4 (1976): 952–958. 971321 10.1016/0006-291x(76)90747-6

[25] O. H. Lowry , N. J. Rosebrough , A. L. Farr , and R. J. Randall , “Protein Measurement With the Folin Phenol Reagent,” Journal of Biological Chemistry 193, no. 1 (1951): 265–275. 14907713

[26] K. J. Livak and T. D. Schmittgen , “Analysis of Relative Gene Expression Data Using Real‐Time Quantitative PCR and the 2^−ΔΔCT^ Method,” Methods 25, no. 4 (2001): 402–408. 11846609 10.1006/meth.2001.1262

[27] S. Aygörmez , M. Makav , M. Kuru , et al., “The Ameliorative Effects of Morin on Colistin‐Induced Kidney Injury in Ovariectomized Rats: Reduces Oxidative Stress, Inflammation Damage, Apoptosis, and Autophagic Death,” Journal of Biochemical Molecular Toxicology 39, no. 11 (2025): e70592. 41183042 10.1002/jbt.70592

[28] M. Kuru , M. Makav , B. B. Kuru , et al., “Hydrogen‐Rich Water Supplementation Improves Metabolic Profile During Peripartum Period in Gurcu Goats and Enhances the Health and Survival of Kids,” Research in Veterinary Science 171 (2024): 105208. 38458045 10.1016/j.rvsc.2024.105208

[29] S. Aygörmez , “Effect of Colistin Injury on Aspartate Aminotransferase, Alanine Aminotransferase and Gamma Glutamyl Transferase Activities in Ovariectomized Rats: Colistin Injury and Enzyme Activities in Rats,” Rats 2, no. 2 (2024): 47–50.

[30] G. Nouioura , T. Kettani , M. Tourabi , et al., “The Protective Potential of *Petroselinumcrispum* (Mill.) Fuss. on Paracetamol‐Induced Hepatio‐Renal Toxicity and Antiproteinuric Effect: A Biochemical, Hematological, and Histopathological Study,” Medicina 59, no. 10 (2023): 1814. 37893532 10.3390/medicina59101814 PMC10608762

[31] O. Rahimi , N. Asadi Louie , A. Salehi , and F. Faed Maleki , “Hepatorenal Protective Effects of Hydroalcoholic Extract of *Solidagocanadensis* L. Against Paracetamol‐Induced Toxicity in Mice,” Journal of Toxicology 2022, no. 1 (2022): 9091605. 36573135 10.1155/2022/9091605 PMC9789909

[32] K. Rahimi , A. Rezaie , Y. Allahverdi , P. Shahriari , and M. Taheri Mirghaed , “The Effects of Alpha‐Pinene Against Paracetamol‐Induced Liver Damage in Male Rats,” Physiological Reports 13, no. 3 (2025): e70227. 39903586 10.14814/phy2.70227 PMC11793005

[33] M. Hafezizadeh , M. Salehcheh , S. Mohtadi , E. Mansouri , and M. J. Khodayar , “Zingerone Effects on Arsenic‐Induced Glucose Intolerance and Hepatotoxicity in Mice via Suppression of Oxidative Stress‐Mediated Hepatic Inflammation and Apoptosis,” Journal of Trace Elements in Medicine and Biology 86 (2024): 127562. 39531827 10.1016/j.jtemb.2024.127562

[34] R. Motamedi , S. Aminzadeh , M. J. Khodayar , L. Khorsandi , and M. Salehcheh , “Protective Effects of Zingerone on Oxidative Stress in Doxorubicin‐Induced Rat Hepatotoxicity,” Reports of Biochemistry and Molecular Biology 12, no. 4 (2024): 575–585. 39086586 10.61186/rbmb.12.4.575 PMC11288236

[35] W. Allam , S. Kasem , S. Mahmoud , A. Abdallah , and M. Hilal , “Experimental Study of the Possible Protective Effect of Alpha‐Lipoic Acid on Paracetamol Induced Oxidative Stress and Hepatic Toxicity in Albino Rats,” Ain Shams Engineering Journal 36, no. 1 (2021): 75–89.

[36] A. Rostami , T. Baluchnejadmojarad , and M. Roghani , “Sinapic Acid Ameliorates Paracetamol‐Induced Acute Liver Injury Through Targeting Oxidative Stress and Inflammation,” Molecular Biology Reports 49, no. 6 (2022): 4179–4191. 35279777 10.1007/s11033-022-07251-1

[37] P. Akbaş , E. Kaya , M. Makav , and G. Yıldız , “Investigation of Antimicrobial and Antioxidant Activities of Chenopodium Album Extracts and Their Effects on Gentamicin Nephrotoxicity in Rats,” Food Science & Nutrition 11, no. 12 (2023): 8121–8130. 38107094 10.1002/fsn3.3733 PMC10724581

[38] C. Kocak , F. E. Kocak , R. Akcilar , et al., “Effects of Captopril, Telmisartan and Bardoxolone Methyl (CDDO‐Me) in Ischemia‐Reperfusion‐Induced Acute Kidney Injury in Rats: An Experimental Comparative Study,” Clinical and Experimental Pharmacology and Physiology 43, no. 2 (2016): 230–241. 26515498 10.1111/1440-1681.12511

[39] C. Kocak , F. E. Kocak , R. Akcilar , et al., “Molecular and Biochemical Evidence on the Protective Effects of Embelin and Carnosic Acid in Isoproterenol‐Induced Acute Myocardial Injury in Rats,” Life Sciences 147 (2016): 15–23. 26820671 10.1016/j.lfs.2016.01.038

[40] S. Zeren , Z. Bayhan , F. E. Kocak , et al., “Gastroprotective Effects of Sulforaphane and Thymoquinone Against Acetylsalicylic Acid–Induced Gastric Ulcer in Rats,” Journal of Surgical Research 203, no. 2 (2016): 348–359. 27363643 10.1016/j.jss.2016.03.027

[41] E. Aktas Senocak , N. Utlu , S. Kurt , S. Kucukler , and F. M. Kandemir , “Sodium Pentaborate Prevents Acetaminophen‐Induced Hepatorenal Injury by Suppressing Oxidative Stress, Lipid Peroxidation, Apoptosis, and Inflammatory Cytokines in Rats,” Biological Trace Element Research 202, no. 3 (2024): 1164–1173. 37393388 10.1007/s12011-023-03755-4

[42] S. Y. Eid , M. F. Koshak , M. E. Elzubier , et al., “Protective Effects of Oral Pharmaceutical Solution of Fucoxanthin Against Paracetamol‐Induced Liver Injury: Modulation of Drug‐Metabolizing Enzymes, Oxidative Stress, and Apoptotic Pathways in Rats,” Drug Development and Industrial Pharmacy 51, no. 4 (2025): 332–343. 39992072 10.1080/03639045.2025.2469808

[43] G. A. Mohamed Kamel , E. Harahsheh , and S. Hussein , “Diacerein Ameliorates Acetaminophen Hepatotoxicity in Rats via Inhibiting HMGB1/TLR4/NF‐κB and Upregulating PPAR‐γ Signal,” Molecular Biology Reports 49, no. 7 (2022): 5863–5874. 35366176 10.1007/s11033-022-07366-5 PMC8975726

[44] M. Elshal and M. E. Abdelmageed , “Diacerein Counteracts Acetaminophen‐Induced Hepatotoxicity in Mice via Targeting NLRP3/Caspase‐1/IL‐1β and IL‐4/MCP‐1 Signaling Pathways,” Archives of Pharmacy & Pharmacology Research 45, no. 3 (2022): 142–158. 10.1007/s12272-022-01373-7 PMC8967791 35244883

[45] S. Küçükler , F. M. Kandemir , S. Özdemir , S. Çomaklı , and C. Caglayan , “Protective Effects of Rutin Against Deltamethrin‐Induced Hepatotoxicity and Nephrotoxicity in Rats via Regulation of Oxidative Stress, Inflammation, and Apoptosis,” Environmental Science and Pollution Research 28, no. 44 (2021): 62975–62990. 34218375 10.1007/s11356-021-15190-w

[46] S. Nithiyanandam and S. E. Prince , “ *Caesalpiniabonducella* Counteracts Paracetamol‐Instigated Hepatic Toxicity via Modulating TNF‐α and IL‐6/10 Expression and Bcl‐2 and Caspase‐8/3 Signalling,” Applied Biochemistry and Biotechnology 195, no. 10 (2023): 6256–6275. 36853441 10.1007/s12010-023-04392-2

[47] R. S. Luty , S. F. Abbas , S. A. Haji , and H. Ridha‐Salman , “Hepatoprotective Effect of Catechin on Rat Model of Acetaminophen‐Induced Liver Injury,” Comparative Clinical Pathology 34 (2025): 705–718.

[48] G. Owusu , M. Antwi‐Adjei , B. Musa , et al., “The Hepatoprotective Effects of Hydroethanolic Stem Bark Extract of *Cleistopholispatens* in Paracetamol‐Induced Hepatotoxicity in Rats,” Journal of Herbs, Spices and Medicinal Plants 31, no. 4 (2025): 559–581.

[49] Z. Liu , F. Yan , H. Zhang , et al., “Zingerone Attenuates Concanavalin A‐Induced Acute Liver Injury by Restricting Inflammatory Responses,” International Immunopharmacology 142 (2024): 113198. 39305891 10.1016/j.intimp.2024.113198

[50] A. H. Chou , C. C. Liao , H. C. Lee , J. T. Liou , and F. C. Liu , “The MAP2K4/JNK/c‐jun Signaling Pathway Plays a Key Role in Dexmedetomidine Protection Against Acetaminophen‐Induced Liver Toxicity,” Drug Design, Development and Therapy 14 (2019): 3887–3898. 10.2147/DDDT.S215473 PMC6861533 31814709

[51] J. Zheng , H. Zhou , T. Yang , et al., “Protective Role of microRNA‐31 in Acetaminophen‐Induced Liver Injury: A Negative Regulator of c‐Jun N‐Terminal Kinase (JNK) Signaling Pathway,” Cellular and Molecular Gastroenterology and Hepatology 12, no. 5 (2021): 1789–1807. 34311140 10.1016/j.jcmgh.2021.07.011 PMC8550922

[52] H. Çelik , S. Kucukler , S. Çomaklı , et al., “Neuroprotective Effect of Chrysin on Isoniazid‐Induced Neurotoxicity via Suppression of Oxidative Stress, Inflammation and Apoptosis in Rats,” Neurotoxicology 81 (2020): 197–208. 33121995 10.1016/j.neuro.2020.10.009

[53] W. P. Jiang , J. S. Deng , S. S. Huang , et al., “Sanghuangporus Sanghuang Mycelium Prevents Paracetamol‐Induced Hepatotoxicity Through Regulating the MAPK/NF‐κB, Keap1/Nrf2/HO‐1, TLR4/PI3K/Akt, and CaMKKβ/LKB1/AMPK Pathways and Suppressing Oxidative Stress and Inflammation,” Antioxidants (Basel) 10, no. 6 (2021): 897. 34199606 10.3390/antiox10060897 PMC8226512

[54] T. Yao , Y. Wu , L. Fu , and L. Li , “Magnolin Ameliorates Acetaminophen‐Induced Liver Injury in Mice via Modulating the MAPK Pathway and Lipid Metabolism,” Toxicology and Applied Pharmacology 497 (2025): 117264. 39952301 10.1016/j.taap.2025.117264

[55] M. C. Indumathi , K. Swetha , K. V. Abhilasha , et al., “Selenium Ameliorates Acetaminophen‐Induced Oxidative Stress via MAPK and Nrf2 Pathways in Mice,” Biological Trace Element Research 202, no. 6 (2024): 2598–2615. 37702962 10.1007/s12011-023-03845-3

[56] C. C. Liao , H. P. Yu , A. H. Chou , H. C. Lee , L. M. Hu , and F. C. Liu , “Gastrodin Alleviates Acetaminophen‐Induced Liver Injury in a Mouse Model Through Inhibiting Mapk and Enhancing Nrf2 Pathways,” Inflammation 45, no. 4 (2022): 1450–1462. 35474551 10.1007/s10753-021-01557-1

[57] J. He , L. Chen , P. Wang , et al., “Network Pharmacology and Experimental Validation of Effects of Total Saponins Extracted From Abrus Cantoniensis Hance on Acetaminophen‐Induced Liver Injury,” Journal of Ethnopharmacology 324 (2024): 117740. 38219885 10.1016/j.jep.2024.117740

[58] M. G. Helali and Y. A. Samra , “Irbesartan Mitigates Acute Liver Injury, Oxidative Stress, and Apoptosis Induced by Acetaminophen in Mice,” Journal of Biochemical and Molecular Toxicology 34, no. 12 (2020): e22447. 31967706 10.1002/jbt.22447

[59] H. E. Kızıl , C. Caglayan , E. Darendelioğlu , et al., “Morin Ameliorates Methotrexate‐Induced Hepatotoxicity via Targeting Nrf2/HO‐1 and Bax/Bcl2/Caspase‐3 Signaling Pathways,” Molecular Biology Reports 50, no. 4 (2023): 3479–3488. 36781607 10.1007/s11033-023-08286-8

[60] S. Hussain , M. Ashafaq , S. Alshahrani , et al., “Cinnamon Oil Against Acetaminophen‐Induced Acute Liver Toxicity by Attenuating Inflammation, Oxidative Stress and Apoptosis,” Toxicology Reports 7 (2020): 1296–1304. 33024703 10.1016/j.toxrep.2020.09.008 PMC7528057

[61] Y. A. Hussein , Y. I. Yahiya , and S. F. Kadhim , “Hepatoprotective, Antioxidant, and Anti‐Inflammatory Properties of Quercetin in Paracetamol Overdose‐Induced Liver Injury in Rats,” Eurasian Journal of Medicine and Oncology 9, no. 2 (2025): 224–233.

[62] B. Eriten , C. Caglayan , C. Gür , S. Küçükler , and H. Diril , “Hepatoprotective Effects of Zingerone on Sodium Arsenite‐Induced Hepatotoxicity in Rats: Modulating the Levels of Caspase‐3/Bax/Bcl‐2, NLRP3/NF‐κB/TNF‐α and ATF6/IRE1/PERK/GRP78 Signaling Pathways,” Biochemical and Biophysical Research Communications 725 (2024): 150258. 38897041 10.1016/j.bbrc.2024.150258

[63] J. Miao , S. Yao , H. Sun , et al., “Protective Effect of Water‐Soluble Acacetin Prodrug on APAP‐Induced Acute Liver Injury Is Associated With Upregulation of PPARγ and Alleviation of ER Stress,” International Journal of Molecular Sciences 24, no. 14 (2023): 11320. 37511082 10.3390/ijms241411320 PMC10380069

[64] Y. Cao , W. He , X. Li , J. Huang , and J. Wang , “Rosiglitazone Protects Against Acetaminophen‐Induced Acute Liver Injury by Inhibiting Multiple Endoplasmic Reticulum Stress Pathways,” BioMed Research International 2022, no. 1 (2022): 6098592. 36588533 10.1155/2022/6098592 PMC9797312

[65] D. Dash and R. K. Koiri , “Therapeutic Modulation of Unfolded Protein Response by Biochemic Tissue Salts in Alcohol‐And Acetaminophen‐Induced Liver Cirrhosis,” Pharmacological Research‐Modern Chinese Medicine 17 (2025): 100710.

[66] S. H. Kim , H. J. Choi , H. Seo , D. Kwon , J. Yun , and Y. S. Jung , “Downregulation of Glutathione‐Mediated Detoxification Capacity by Binge Drinking Aggravates Acetaminophen‐Induced Liver Injury Through IRE1α ER Stress Signaling,” Antioxidants 10, no. 12 (2021): 1949. 34943052 10.3390/antiox10121949 PMC8750905

[67] N. Demirtas , B. S. Mazlumoglu , N. A. Celep , E. Cadirci , Z. Halici , and S. S. Palabiyik‐Yucelik , “Epigallocatechin‐3‐Gallate Provides Hepatoprotection Through Endoplasmic Reticulum Stress/TXNIP/NLRP3 Axis in Paracetamol‐Induced Acute Liver Injury,” Basic & Clinical Pharmacology & Toxicology 138, no. 1 (2026): e70155. 41340591 10.1111/bcpt.70155

[68] H. Zou , J. Sun , B. Wu , et al., “Effects of Cadmium and/or Lead on Autophagy and Liver Injury in Rats,” Biological Trace Element Research 198, no. 1 (2020): 206–215. 32006201 10.1007/s12011-020-02045-7

[69] M. Jalouli , A. Mofti , Y. A. Elnakady , et al., “Allethrin Promotes Apoptosis and Autophagy Associated With the Oxidative Stress‐Related PI3K/AKT/mTOR Signaling Pathway in Developing Rat Ovaries,” International Journal of Molecular Sciences 23, no. 12 (2022): 6397. 35742842 10.3390/ijms23126397 PMC9224321

[70] F. Cakmak , S. Kucukler , C. Gur , et al., “Provides Therapeutic Effect by Attenuating Oxidative Stress, Inflammation, Endoplasmic Reticulum Stress, Autophagy, Apoptosis, and Oxidative DNA Damage in Testicular Toxicity Caused by Ifosfamide in Rats,” Iranian Journal of Basic Medical Sciences 26, no. 10 (2023): 1227. 37736509 10.22038/IJBMS.2023.71702.15580 PMC10510477

[71] S. Liu , J. Ren , S. Liu , et al., “Resveratrol Inhibits Autophagy Against Myocardial Ischemia‐Reperfusion Injury Through the DJ‐1/MEKK1/JNK Pathway,” European Journal of Pharmacology 951 (2023): 175748. 37149277 10.1016/j.ejphar.2023.175748

[72] Y. Zhu , S. Zhang , Y. Shao , et al., “Regulatory Role of Oxidative Stress in Retrorsine–Induced Apoptosis and Autophagy in Primary Rat Hepatocytes,” Ecotoxicology and Environmental Safety 279 (2024): 116515. 38810283 10.1016/j.ecoenv.2024.116515

[73] K. K. Zhang , H. Wang , D. Qu , et al., “Luteolin Alleviates Methamphetamine‐Induced Hepatotoxicity by Suppressing the p53 Pathway‐Mediated Apoptosis, Autophagy, and Inflammation in Rats,” Frontiers in Pharmacology 12 (2021): 641917. 33679421 10.3389/fphar.2021.641917 PMC7933587

[74] Y. Guo , Y. Yuan , R. Wang , et al., “Monocrotaline‐Mediated Autophagy via Inhibiting PI3K/AKT/mTOR Pathway Induces Apoptosis in Rat Hepatocytes,” Frontiers in Pharmacology 15 (2024): 1499116. 39494350 10.3389/fphar.2024.1499116 PMC11527718

[75] Z. Li , M. Liu , J. Li , G. Yan , and X. Xu , “Diosmetin Alleviates AFB1‐Induced Endoplasmic Reticulum Stress, Autophagy, and Apoptosis via PI3K/AKT Pathway in Mice,” Ecotoxicology and Environmental Safety 292 (2025): 117997. 40037078 10.1016/j.ecoenv.2025.117997

[76] E. M. Samy and E. A. Shaaban , “Liraglutide Ameliorates Gamma Radiation‐Induced Hepatic Damage in Rats: The Role of an Autophagy Flux Activation via LKB1/AMPK/mTOR Axis,” Archives of Medical Research 57, no. 2 (2026): 103296. 40913889 10.1016/j.arcmed.2025.103296

[77] F. M. Kandemir , S. Kucukler , E. Eldutar , C. Caglayan , and İ. Gülçin , “Chrysin Protects Rat Kidney From Paracetamol‐Induced Oxidative Stress, Inflammation, Apoptosis, and Autophagy: A Multi‐Biomarker Approach,” Scientia pharmaceutica 85, no. 1 (2017): 4. 28134775 10.3390/scipharm85010004 PMC5388142

[78] J. Zhao , K. Ding , M. Hou , et al., “ *Schisandrachinensis* Essential Oil Attenuates Acetaminophen‐Induced Liver Injury Through Alleviating Oxidative Stress and Activating Autophagy,” Pharmaceutical Biology 60, no. 1 (2022): 958–967. 35588406 10.1080/13880209.2022.2067569 PMC9122381

[79] M. Ileriturk and F. M. Kandemir , “Carvacrol Protects Against λ‐Cyhalothrin‐Induced Hepatotoxicity and Nephrotoxicity by Modulating Oxidative Stress, Inflammation, Apoptosis, Endoplasmic Reticulum Stress, and Autophagy,” Environmental Toxicology 38, no. 7 (2023): 1535–1547. 36947485 10.1002/tox.23784

[80] B. Varışlı , C. Caglayan , F. M. Kandemir , et al., “Chrysin Mitigates Diclofenac‐Induced Hepatotoxicity by Modulating Oxidative Stress, Apoptosis, Autophagy and Endoplasmic Reticulum Stress in Rats,” Molecular Biology Reports 50, no. 1 (2023): 433–442. 36344803 10.1007/s11033-022-07928-7

[81] M. Talukder , S. S. Bi , M. W. Lv , J. Ge , C. Zhang , and J. L. Li , “Involvement of the Heat Shock Response (HSR) Regulatory Pathway in Cadmium‐Elicited Cerebral Damage,” Environmental Science and Pollution Research 30, no. 48 (2023): 106648–106659. 37730984 10.1007/s11356-023-29880-0

[82] Y. Song , X. Song , M. Wu , et al., “The Protective Effects of Melatonin on Survival, Immüne Response, Digestive Enzymes Activities and Intestinal Microbiota Diversity in Chinese Mitten Crab (*Eriocheirsinensis*) Exposed to Glyphosate,” Comparative Biochemistry and Physiology Part C: Toxicology & Pharmacology 238 (2020): 108845. 32777465 10.1016/j.cbpc.2020.108845

[83] C. Adiguzel , H. Karaboduk , F. G. Apaydın , and Y. Kalender , “Effects of Quercetin on Palladium Chloride‐Induced Endoplasmic Reticulum Stress, Inflammation, Oxidative Stress, and Apoptosis in Hepatorenal Tissues,” Microscopy and Microanalysis 31, no. 4 (2025): ozaf077. 40875569 10.1093/mam/ozaf077

[84] N. M. Mesalam , M. A. Ibrahim , M. R. Mousa , and N. M. Said , “Selenium and Vitamin E Ameliorate Lead Acetate‐Induced Hepatotoxicity in Rats via Suppression of Oxidative Stress, mRNA of Heat Shock Proteins, and NF‐kB Production,” Journal of Trace Elements in Medicine and Biology 79 (2023): 127256. 37442019 10.1016/j.jtemb.2023.127256

